# Cancer surgery during COVID increased the patient mortality and the transmission risk to healthcare workers: results from a retrospective cohort study (NCT05240378)

**DOI:** 10.1186/s12957-022-02761-5

**Published:** 2022-09-20

**Authors:** Kishan Soni, J. F. Neville, Roli Purwar, Tarun Kumar, Ghanshyam Yadav, Nimisha Verma, Manoj Pandey

**Affiliations:** 1 Department of Surgical Oncology, Institute of Medical Sciences, Banaras Hindu University, Varanasi, India; 2 Department of Anesthesia, Institute of Medical Sciences, Banaras Hindu University, Varanasi, India

**Keywords:** Cancer, COVID-19 infection, SARS-CoV-2, Morbidity, Mortality, Complications

## Abstract

**Background:**

India encountered two waves of COVID-19 pandemic with variability in its characteristics and severity. Concerns were raised over the safety of treatment, and higher morbidity was predicted for oncological surgery. The present study was conducted to evaluate and compare the rate of morbidity and mortality in patients undergoing curative surgery for cancer before and during the COVID-19 pandemic.

**Method:**

The prospectively obtained clinical data of 1576 patients treated between April 2019 and May 2021 was reviewed; of these, 959 patients were operated before COVID-19 and 617 during the pandemic. The data on complications, deaths, confirmed or suspected COVID-19 cases, and COVID-19 infection among health workers (HCW) was extracted.

**Results:**

A 35% fall in number of surgeries was seen during the COVID period; significant fall was seen in genital and esophageal cancer. There was no difference in postoperative complication; however, the postoperative mortality was significantly higher. A total of 71 patients had COVID-19, of which 62 were preoperative and 9 postoperative, while 30/38 healthcare workers contracted COVID-19, of which 7 had the infection twice and 3 were infected after two doses of vaccination; there was no mortality in healthcare workers.

**Conclusion:**

The present study demonstrates higher mortality rates after surgery in cancer patients, with no significant change in morbidity rates. A substantial proportion of HCWs were also infected though there was no mortality among this group. The results suggest higher mortality in cancer patients despite following the guidelines and protocols.

**Supplementary Information:**

The online version contains supplementary material available at 10.1186/s12957-022-02761-5.

## Introduction

In December 2019, the outbreak of SARS-CoV-2 (severe acute respiratory syndrome coronavirus 2) virus was first diagnosed, and it spreads worldwide to become a pandemic [1]. The disease was initially reported in Wuhan, China, with causative agent being identified as a novel enveloped betacoronavirus; it has infected people residing all over the world, with about 579,092,623 confirmed cases and 6,407,556 deaths worldwide as on August 1, 2022 [2].

Despite its first identification nearly 3 years ago, we are still in the middle of pandemic with waves after waves infecting people. The vaccination program in India was started in January 2021 [3], and a significant population has been vaccinated. However, the infections are identified in vaccinated population as well, though they are asymptomatic or mild, more so in healthcare workers (HCW) where exposure rates are high [4]. India, being the developing country, was seriously affected with this novel coronavirus disease (SARS-CoV-2) outbreak that placed unprecedented demands on its healthcare facilities [5, 6]. Like other countries, India too encountered the two-wave pattern of COVID-19 during the period of 2020 and 2021, with the first wave in winter of 2020 and the second wave in summer of 2021 [7]. Between these two periods, the traits of the disease like severity, effects, characteristics of the virus, morbidity, and mortality with this pandemic varied. A number of mutations have been reported in the virus that contributed to changed symptom profile and mortality pattern. After the USA, India has highest number of confirmed cases of SARS-CoV-2 infection, with peak rise reported in May 2021 [2]. Regarding mortality rate, India is reported as third place after the USA and Brazil [2].

Because of complex mode of transmission and lack of an established treatment, SARS-CoV-2 infection posed a global challenge [8]. This challenge was more damaging for low- and middle-income countries like India, due to their compromised healthcare system, shortage of skilled health workers, lack of disease awareness, and occurrence of comorbidities or infections along with malnutrition in infected patients [9].

In India, to control the spread of the pandemic, various restrictions on travel were posed like limitation in the public transport facilities and ban on inter- and intrastate travel. Due to these restrictions and fear of getting infected with SARS-CoV-2 in hospitals, the access to healthcare facilities by patients was extremely affected [5]. Furthermore, many hospitals were giving priority to only essential procedures while deferring various nonessential services [10]. The patients suffering with cancer were apprehensive to report to hospitals as they were also not excluded from these restriction guidelines. Even though most centers like ours continued to provide services, there were limitations like availability of ICU bed or ventilators that precluded many surgeries; some of the cancer hospitals in our region were converted to COVID-19 hospitals. This caused a substantial delay in the treatment for patients suffering from cancer. For a cancer center, the priority is to deliver an early treatment to a patient suffering with cancer, and imposition of restriction and availability posted a big challenge to meet the goals.

Although cancer is the second principal cause of mortality worldwide, however, limited data was available on cancer patients undergoing treatment during COVID-19 when this study was planned. Most of the data that was available showed an increased morbidity and mortality with increased ICU admissions. The present study is a retrospective analysis of a prospective database from a tertiary referral and teaching center where the cancer services continued despite constraints; apart from patient mortality and morbidity, the data on COVID-19 infections in patients before or after surgery and infections in the HCWs exposed to these patients is also reported upon.

## Materials and method

The present study is a retrospective analysis of prospective electronic database. The study reports on cancer patients operated between May 2020 and May 2021 during COVID and compares the data from preceding pre-COVID year (April 2019–April 2020) at our center. Essentially, the pre-COVID-19 data is until 18 March 2020, as on this day the first lockdown was declared in India and our services were also stopped until 18 May, 2020, when they were restarted and remain uninterrupted till date. All patients who underwent surgery during these two time periods were included in the study. The end points recorded and reported are as follows: (a) number of minor and major postoperative complications using Clavien-Dindo classification, (b) pre- and post-COVID period SARS-CoV-2 infections in patients and healthcare workers (HCW), (c) 30-day postoperative mortality. The study was approved by the Institute Ethics Committee, Institute of Medical Sciences, Banaras Hindu University, Varanasi, India, and is registered at clinicaltrials.gov with registration number NCT05240378 (available at https://clinicaltrials.gov/show/NCT05240378). The work has been reported in line with the STROCSS guidelines [11].

When the services were resumed in May 2020, a strict COVID cancer surgery protocol was adopted by the department approved by the institution and was followed by all [10]. This essentially consisted of COVID RT-PCR testing and chest CT for all patients before admission, holding the patients in holding area until reports were available and then moving them to wards, increasing the distance between patients in ward, use of full PPE kits by HCW, use of non-centralized air-conditioners with filters, using double filters in anesthesia machines and discarding circuits after use, reduction in aerosol generating procedures, operating on ASA grades 1–3 patients, etc. [10].

The data obtained was analyzed using IBM SPSS Statistics for Windows, version 21.0 (IBM Corp., Armonk, NY, USA). Chi-square test was used to calculate the probability value between groups, and a value of < 0.05 was considered significant.

## Results

A total of 1576 patients were included in the study, of which 959 patients were operated pre-COVID and 617 during COVID-19 pandemic period. A fall of 35% in case number was observed between two time periods. Of all types of cancers, head and neck cancers were most common, followed by gastrointestinal (GI) and breast cancers in both pre-COVID and during COVID period; however, the difference between two periods was statistically significant (*p*-value < 0.05). The maximum change was observed in other cancers, head neck cancers and breast cancers (Table 1). The patient demography like stage of disease and comorbidities are described in Table 2. Except for more locally advanced cases being operated in COVID period, all other characteristics were similar in both groups.

**Table 1:** Site-wise distribution of cases before and during COVID period

Site of cancer	Group A before COVID period (April 2019–April 2020)		Group B COVID period (May 2020–May 2021)		Change (%) for site between two time periods
	Number	Percentage	Number	Percentage	
**Breast**	180	18.76	105	17.01	-26.32
**Others**	70	7.29	37	5.99	-30.84
**Thorax**	5	0.52	4	0.64	-11.11
**GIT**	207	21.58	151	24.47	-15.64
**Head and neck**	340	35.45	197	31.92	-26.63
**Gynecology**	125	13.03	77	12.47	-23.76
**Male genital and urinary system**	32	3.33	24	3.88	-14.29
**Total**	959	100	617	100	-21.70
**Chi-square test**	11.87				
***p*-value**	0.044*				

*p-value < 0.05 is significant

**Table 2:** Baseline characteristics of two groups

Variable	Group A Before COVID period (April 2019–April 2020)		Group B COVID period (May 2020–May 2021)		*p*-value
Age (mean)	51.18	-	48.33	-	< 0.05
**Gender**					
Female	512	53%	335	54%	0.72
Male	447	47%	282	46%	
**Comorbidity**					
Diabetes	42	4.3%	31	5%	0.8
Hypertension	77	8%	49	7.9%	
Other	26	2.7	15	2.4%	
**ECOG**					
1	959	100%	617	100	NC
**T stage**					
1	34	3.5%	9	1.4%	0.04
2	248	25.8%	146	23.6%	
3	341	35.5%	242	39.3%	
4	336	35%	220	35.6%	
**N stage**					
0	440	45.8%	246	39.8%	0.01
N+	519	54.2%	371	60.2%	
**M stage**					
0	959	100%	617	100%	NC
**Neoadjuvant treatment**					
Yes	289	30%	184	29.8%	0.89
No	670	70%	433	70.2%	

*NC* Not calculated

COVID-19 infection was observed in 62 (10.05%) patients preoperative and 9 (1.45%) patients postoperatively; majority of these patients were of head and neck and GI cancers. The difference was statistically significant (*p* < 0.05) (Table 3). It was observed that the postoperative mortality was 2.26% during COVID period, compared to 0.93% in pre-COVID period; the difference was statistically significant (*p* < 0.05). Out of 14 deaths in COVID period, 7 were suspected to be due to COVID-19 infection; of these, 4 were confirmed by RT-PCR, while 3 showed atypical pneumonia on HRCT suggestive of COVID-19 infection with negative RT-PCR (Table 4). The postoperative complications other than COVID infection remained the same during both time periods (*p* > 0.05). In both periods, around 12% patients had postoperative complications; of these, 8 to 9% had minor, and 3% had major complications (Table 5).

**Table 3:** Distribution of cases with COVID-19 infection

Site of cancer	Infected during preoperative period (group A)		Infected during postoperative period (group B)	
	Number	Percentage	Number	Percentage
**Breast**	11	1.78	0	0
**Others**	4	0.64	0	0
**Thorax**	1	0.16	0	0
**GIT**	10	1.62	5	0.81
**Head and neck**	21	3.40	4	0.64
**Gynecology**	12	1.94	0	0
**Male genital and urinary system**	3	0.48	0	0
**Total**	62	10.04	9	1.45
**Chi-square**	9.0			
***p*-value**	0.002*			

*p-value < 0.05 is significant

**Table 4:** Postoperative mortality before and during COVID period

Site	Before COVID period (April 2019–April 2020) (group A) (***N*** = 959)		COVID period (May 2020–May 2021) (group B) (***N*** = 617)	
	Number	Percentage	Number	Percentage
**Breast**	0	0	0	0
**Others**	0	0	1	0.16
**Thorax**	2	0.2	0	0
**GIT**	5	0.52	7 (3^a^ + 2^b^)	1.13
**Head and neck**	1	0.1	5 (1^a^ + 1^b^)	0.81
**Gynecology**	0	0	1	0.16
**Male genital and urinary system**	1	0.1	0	0
**Total**	9	0.93	14	2.26
**Chi-square**	2.16			
***p*-value**	0.04*			

*p-value < 0.05 is significant. Mortality during COVID period is 14, out of which 7 is due to COVID (4 were ^a^confirmed cases, and 3 had ^b^atypical pneumonia on HRCT suggestive of COVID infection)

**Table 5:** Postoperative complications based on Clavien-Dindo classification before and during COVID period (excluding COVID infection)

Site	Group A Before COVID period (April 2019–April 2020)			Group B COVID period (May 2020–May 2021)		
	Minor	Major	Total	Minor	Major	Total
**Breast**	16	0	16	9	0	9
**Others**	10	4	14	5	2	7
**Thorax**	1	0	1	1	0	1
**GIT**	8	18	26	4	16	20
**Head and neck**	36	10	46	18	6	24
**Gynecology**	12	0	12	8	0	8
**Male genital and urinary system**	7	0	7	6	0	6
**Total**	90/9.38	32/3.33	122/12.7	51/8.26	24/3.88	75/12.16
**Before vs during COVID**	Minor	*χ*^2^: 10.99; *p*-value: 0.061*				
	Major	*χ*^2^: 5.002; *p*-value: 0.07*				
	Total	*χ*^2^: 12.031; *p*-value: 0.057*				

*p-value > 0.05 is insignificant

**Table 6:** Infections in healthcare workers during COVID period

	Total	COVID positive	Percentage
**Doctors**	18	16 (4^a^ + 2^b^)	88.89
**Nurses/paramedics**	20	14 (3^a^ + 1^b^)	70
**Total**	38	30	78.95

Seven were ^a^infected twice, and 3 were ^b^infected after vaccination

Out of 38 staff members that included doctors, nurses, and paramedics, 30 (78.95%) were infected with SARS-CoV-2. The proportion was 88.89% among doctors and 70% among nurses. Seven of the healthcare workers were infected twice, and 3 were infected after vaccination (Tables 5 and 6). There were no cases of severe COVID-19 or death among HCW.

## Discussion

On 18 March 2020, the routine healthcare services including cancer surgeries were halted, and hospital was dealing with COVID-19-infected patients, and only emergency services were running; the hospital was converted to level 3, COVID-19 hospital. On 28 April 2020, the Department of Surgical Oncology started out-patient services, and from May 19, 2020, the operation rooms (ORs) were permitted to open with a definitive protocol in place [10].

A number of articles and a few protocols have been published on cancer and COVID; however, there are only a few articles reporting on the data on the management of cancer patients [12, 13]. There are two systematic review and meta-analysis; both of these looked at mortality in patients with COVID-19 infection undergoing surgery and reported it to be as high as 20% with ICU admission rate of 15% [14, 15]. A higher risk of mortality after surgery was shown in hospitals with more than 25% of patients with COVID-19 [16], while others have reported no increase in mortality [17]. Most studies reporting worst outcome reported late presentation or delay as the cause of increased mortality [18, 19]. The presence of perioperative COVID-19 has also been found to be associated with higher mortality and morbidity [20]. However, the data on surgery on COVID-19 RTPCR-negative cancer patients undergoing surgery under strict protocol has not been reported before.

The present study demonstrates no changes in the morbidity and significantly high mortality in cancer patients before and during the COVID-19 pandemic besides demonstrating an increase in COVID-19 infection rates and the higher risk of infection to HCW. Although earlier studies reported the impact of COVID pandemic on rate of complications, mortality, delayed reporting, and management of cancer patients [21, 22, 23], however, data on infection of operating surgeons and operation room nurses and other healthcare workers exposed to SARS CoV2 is lacking. Furthermore, the present retrospective analysis of prospectively maintained database is the first cohort study that compares the morbidity and mortality of cancer patients before and during the COVID phase, while most other studies reported only on patients operated during COVID.

In present study, a fall of 35% in number of surgeries performed was observed during COVID period which is similar to other studies that too demonstrated a reduced footfall during COVID [24]. Many of the reasons for this at our center are discussed in our earlier publication [10] and hence are not repeated here.

The postoperative complications other than COVID infection remained the same during both time periods (*p*-value > 0.05). In contrast to our study, Zhang L. et al. [15] reported that COVID-19-infected cancer patients have a high risk of poor clinical outcomes, severe event, and mortality. Similarly, Al-Quteimat O. M. et al. [21] found that the cancer history conferred the highest risk for severe complications and was correlated with poorer outcomes from COVID-19. This was due to the fact that the immunosuppressed status of some cancer patients (whether caused by the disease itself or the treatment) increases their risk of infection compared with the general population.

We observed a significant change associated with mortality of cancer patients. The rate of mortality significantly increased during COVID-19 period. Out of 14 deaths in COVID period, 7 were due to COVID infection. This is perhaps due to the reasons that patients suffering with cancer are more prone to mortality if got infected with SARS-CoV-2-virus. Other than that, the mortality increase may have been due to nonavailability of ICU and ventilators, delay in start of the treatment after developing pneumonia, or due to affected healthcare services with other experts not being available, financial issues, nonavailability and shortage of medications due to lockout, hampered transport services, etc. [24, 25]. Previous studies have also shown that the cancer patients are more vulnerable to severe COVID-19 infections with almost doubling of the hazards [26–29]. Strict cross-infection prevention protocols were followed to prevent the chances of COVID infection among admitted cancer patients, and yet, they were not successful probably due to high infection rates in population and community transmission of the virus.

Though the main aim was to deliver the utmost care to cancer-affected patients by following all precautions and COVID safety protocols, so as to reduce the COVID-19 infection and mortality in the cancer patients, however, we observed that around 78% healthcare workers got infected with COVID. As there was a protocol in place in the hospital and contact was minimized with infected patients with hospital divided in red, yellow, and green zones with no intermixing of staff and patients, it is assumed that the infections were acquired at home and in the community and not in the hospital. The hospital has appointed a microbiologist as infection control officer who evaluated all staff members after each contact, and HCW were required to fill a predesigned form detailing the contact and personal protective measures that they were using at the point of contact. Based on this evaluation, the risk was categorized as low, medium, and high. All HCW that were considered to have high and medium risk were advised to stay home in isolation and get the RT-PCR between 3 and 5 days after the contact. If RT-PCR was negative, they were allowed to resume duties, while those positive were home isolated for 13 days in the beginning of COVID and later for 9 days as per ICMR guidelines.

The biggest limitation of our study is that it is limited to a specific geographical area, and results cannot be applicable to whole country or the world, as each hospital and city had different limitation to its working, and generalization is not possible until the data across the world is pooled. Even though the database is prospective, the analysis is retrospective; hence, selection bias is present, and reasons for this bias have been discussed above. Despite its limitation, the study clearly demonstrates a statistically significant increase in mortality among RT-PCR negative patients, undergoing elective cancer surgery during COVID period, even though the postoperative infection rates during hospitalization were low.

## Conclusion

The present study demonstrates that despite low postoperative infection from COVID-19 and adherence to the guidelines and protocol, the mortality is significantly high in RT-PCR-negative cancer patients undergoing elective surgery, while the morbidity remains the same. The study further shows high risk of COVID-19 infection among healthcare workers; however, the infections were mild to moderate, and none required ICU admission.

## Supplementary Information

**Additional file 1.** The STROCSS 2021 Guideline.

## Data Availability

The data will be made available on reasonable request.

## References

[1] WHO director-general’s opening remarks at the media briefing on COVID-19 - 11 March 2020. https://www.who.int/director-general/speeches/detail/who-director-general-s-opening-remarks-at-the-media-briefing-on-covid-19%2D%2D-11-march-2020 [Last Accessed on 7 Aug 2022].

[2] WHO coronavirus (COVID-19) dashboard. https://covid19.who.int/?adgroupsurvey={adgroupsurvey}&gclid=Cj0KCQjworiXBhDJARIsAMuzAuwiRxSuAfPBNmWtGBde2g0j0dwVklsjjRPN6drHibgGpGW3n3QEnmMaAjVzEALw_wcB [Last Accessed 7 Aug 2022].

[3] The worlds largest vaccination drive. Ministry of health and family welfare, Government of India https://www.mohfw.gov.in/pdf/COVIDVaccinationBooklet14SEP.pdf [Last Accessed 7 Aug 2022].

[4] Kaur U, Bala S, Joshi A, NTS R, Japur C, Chauhan M, Pedapanga N, Kumar S, Mukherjee A, Mishra V, Talda D, Singh R, Gupta RK, Yadav AK, Rana PJ, Srivastava J, Bhat KS, Singh A, NK PG, Pandey M, Patwardhan K, Kansal S, Chakrabarti SS (2022). Persistent health issues, adverse events, and effectiveness of vaccines during the second wave of COVID-19: a cohort study from a tertiary hospital in North India. Vaccines (Basel).

[5] Gupta N, Chauhan AS, Prinja S, Pandey AK (2021). Impact of COVID-19 on outcomes for patients with cervical cancer in India. JCO Global Oncol.

[6] Iftimie S, López-Azcona AF, Vallverdú I, Hernández-Flix S, de Febrer G, Parra S (2021). First and second waves of coronavirus disease-19: a comparative study in hospitalized patients in Reus, Spain. PLoS One..

[7] Walker PG, Whittaker C, Watson OJ, Baguelin M, Winskill P, Hamlet A (2020). The impact of COVID-19 and strategies for mitigation and suppression in low-and middle-income countries. Science..

[8] Bong CL, Brasher C, Chikumba E, McDougall R, Mellin-Olsen J, Enright A. The COVID-19 pandemic: effects on low-and middle-income countries. Anesth Analg. 2020;131(1):86–92. 10.1213/ANE.0000000000004846. 10.1213/ANE.0000000000004846 PMC7173081 32243287

[9] The Lancet Oncology (2020). COVID-19: global consequences for oncology. Lancet Oncol.

[10] Batra TK, Tilak MR, Pai E, Verma N, Gupta BK, Yadav G (2021). Increased tracheostomy rates in head and neck cancer surgery during the COVID-19 pandemic. Int J Oral Maxillofac Surg.

[11] Agha R, Abdall-Razak A, Crossley E, Dowlut N, Iosifidis C, Mathew G, For The STROCSS Group (2019). The STROCSS 2019 guideline: strengthening the reporting of cohort studies in surgery. Int J Surg.

[12] Madan A, Siglin J, Khan A (2020). Comprehensive review of implications of COVID-19 on clinical outcomes of cancer patients and management of solid tumors during the pandemic. Cancer Med..

[13] Chakraborty M, Pandey M (2020). Caring for cancer patients in the Covid pandemic: choosing between the devil and deep sea. World J Surg Onc.

[14] Abate SM, Mantefardo B, Basu B (2020). Postoperative mortality among surgical patients with COVID-19: a systematic review and meta-analysis. Patient Saf Surg.

[15] Zhang L, Zhu F, Xie L, et al. Clinical characteristics of COVID-19-infected cancer patients: a retrospective case study in three hospitals within Wuhan, China. Ann Oncol. 2020; Available at: https://www.annalsofoncology.org/article/S0923-7534(20)36383-3/fulltext. 10.1016/j.annonc.2020.03.296 PMC7270947 32224151

[16] Glance LG, Dick AW, Shippey E, McCormick PJ, Dutton R, Stone PW, Shang J, Lustik SJ, Lander HL, Gosev I, Joynt Maddox KE (2022). Association between the COVID-19 pandemic and insurance-based disparities in mortality after major surgery among US adults. JAMA Netw Open..

[17] Russell B, Moss C, Monroy-Iglesias M, Roberts G, Dickinson H, Haire K, et al. Radical cancer treatment is safe during COVID-19: the real-world experience of a large London-based Comprehensive Cancer Centre during the first wave. Br J Cancer. 2022:1–7. 10.1038/s41416-022-01909-0 Epub ahead of print. PMID: 35840733; PMCID: PMC9284490. 10.1038/s41416-022-01909-0 PMC9284490 35840733

[18] Fotopoulou C, Khan T, Bracinik J, Glasbey J, Abu-Rustum N, Chiva L, Fagotti A, Fujiwara K, Ghebre R, Gutelkin M, Ng J, Pareja R, Seenivasagam RK, Sehouli J, Surappa S, Bhangu A, Leung E, Sundar S, Konney TO (2022). CovidSurg Gynecological cancer collaborators. Outcomes of gynecological cancer surgery during the COVID-19 pandemic: an international, multicenter, prospective CovidSurg-GO Cancer study. Am J Obstet Gynecol.

[19] Ma J, Zhu C, Li W, Qiu Z, Yang J, Ge L, Da M (2022). The effect of delayed oncology surgery on survival outcomes for patients with gastric cancer during the COVID-19 pandemic: evidence-based strategies. Front Oncol..

[20] COVIDSurg Collaborative (2020). Mortality and pulmonary complications in patients undergoing surgery with perioperative SARS-CoV-2 infection: an international cohort study. Lancet.

[21] Al-Quteimat OM, Amer AM (2020). The impact of the COVID-19 pandemic on cancer patients. Am J Clin Oncol..

[22] Wang H, Zhang L (2020). Risk of COVID-19 for patients with cancer. Lancet Oncol..

[23] Dai M, Liu D, Liu M, Zhou F, Li G, Chen Z, Zhang Z, You H, Wu M, Zheng Q (2020). Patients with cancer appear more vulnerable to SARS-CoV-2: a multicenter study during the COVID-19 outbreak. Cancer Discov..

[24] Das R, Nahak SR, Parija J (2021). Optimising cancer surgery during COVID-19: experience of tertiary cancer centre in eastern India. Indian J Gynecol Oncolog.

[25] Choi JY, Park IJ, Lee HG, Cho E, Kim YI, Kim CW, Yoon YS, Lim SB, Yu CS, Kim JC (2021). Impact of the COVID-19 pandemic on surgical treatment patterns for colorectal cancer in a tertiary medical facility in Korea. Cancers (Basel)..

[26] Jee J, Foote MB, Lumish M, Stonestrom AJ, Wills B, Narendra V, et al. Chemotherapy and COVID-19 outcomes in patients with cancer. J. Clin. Oncol. 2020; JCO.20.01307. 10.1200/JCO.20.01307 PMC7571792 32795225

[27] Yu J, Ouyang W, Chua MLK, Xie C. SARS-CoV-2 transmission in patients with cancer at a tertiary care hospital in Wuhan, China. JAMA Oncol. 2020. 10.1001/jamaoncol.2020.0980 PMC7097836 32211820

[28] Lei S, Jiang F, Su W, Chen C, Chen J, Mei W, Zhan LY, Jia Y, Zhang L, Liu D, Xia ZY, Xia Z (2020). Clinical characteristics and outcomes of patients undergoing surgeries during the incubation period of COVID-19 infection. EClinicalMedicine..

[29] Zoilo M, Javier O, Aurema O, Sebastiano B, Sebastian V (2021). Postoperative complications and mortality following emergency digestive surgery during the COVID-19 pandemic. Medicine.

